# The Diagnosis in a Case of Pernicious Anæmia

**Published:** 1909-10-09

**Authors:** C. O. Hawthorne

**Affiliations:** Examiner in Medicine and Clinical Medicine in the University of Glasgow; Physician to the North-West London Hospital and to the Central London Ophthalmic Hospital


### Hospital Clinics.

### THE DIAGNOSIS IN A CASE OF PERNICIOUS ANÆMIA.

By C. O. HAWTHORNE, M.D., M.R.C.P.; Examiner in Medicine and Clinical Medicine in the University of Glasgow; Physician to the North-West London Hospital and to the Central London Ophthalmic Hospital.

(A Lecture delivered at the Medical Graduates' College and Polyclinic.)

Gentlemen,—The patient to whose case I wish to direct your attention this afternoon is a woman of 52 years. Some 15 to 18 months ago she began to complain of general weakness and of breathlessness on exertion, and these symptoms have gradually become more troublesome. For several months, too, there has been œdematous swelling of the lower limbs, and neither tonics, given with the view of improving the general health, nor diuretics, ordered in the hope of removing the dropsy, have produced any substantial benefit. The œdema has, I understand, been considered a rather disconcerting and mysterious occurence, seeing that no organic disease of any of the thoracic or abdominal organs, and competent to explain the existence of the dropsy, can be discovered. Thus the case has been regarded as somewhat of a diagnostic puzzle, inasmuch as, though œdema of the lower limbs is a conspicuous symptom, none of the usual causes of this condition appears to be present. The fact is, I suppose, that dropsy of the lower limbs is so frequently the result of disease of the heart, kidneys, or other organs, that its existence at once arouses suspicion in these directions, and when physical examination in an individual case shows this suspicion to be baseless, there is apt to be a sense of surprise. The surprise, however, ought only to be momentary, as there are well-established causes of œdema of the lower limbs other than organic affections of certain of the thoracic and abdominal viscera. Thus some of you may recall a series of cases of peripheral neuritis in chronic alcoholic patients and attended by œdema of the feet and legs which I demonstrated in this classroom last year. The suggestion in such cases is that the poison, by paralysing the vaso-motor nerves, upsets the balance of the tissue circulation and so favours the accumulation of fluid in the tissue spaces. Whatever be the exact explanation, the fact is beyond dispute, that peripheral neuritis, as well as renal and cardiac disease, may be associated with subcutaneous œdema. Another cause of such œdema is found in various diseases which depreciate the quality of the blood. Here there is probably both malnutrition of the capillary blood-vessels, allowing increased transudation of fluid, and malnutrition of the cardiac muscle, with, as a consequence, a sluggish circulation in the capillaries and veins; and both these agencies will be factors in the production of the dropsy. Examples of the action of defective blood-quality as a clinical cause of œdema are sometimes seen in chlorosis and in the cachexia produced by malignant disease. But probably the most severe manifestations of œdema due to this cause occur in such blood diseases as pernicious anæmia, leucocythæmia, and other conditions of a more or less similar nature. Therefore both peripheral neuritis and various abnormal blood conditions must be recognised among the possible causes of œdema of the lower limbs, and when, as in the present instance, clinical examination shows, together with such œdema, the absence of visceral disease, these causes ought to come prominently into view. Had this truth been borne in mind, it is probable that the correct diagnosis in the present case would have been reached at a much earlier stage. For, just as examination of the patient shows the absence of distinctive evidence of visceral disease, it also shows the absence of all signs of peripheral neuritis. Hence, by the method of exclusion, all positive facts apart, the inquiry is naturally directed to the condition of the blood as the probable source where an explanation of the œdema is to be found.

Another aid to clinical investigation which might with advantage have been laid under contribution in the present patient at an earlier stage is the ophthalmoscope. Immediately this method of observation is employed the diagnostic position of the case is greatly improved. For, scattered widely over each fundus oculi are numerous retinal hæmorrhages, and multiple retinal hæmorrhages having a bilateral distribution carry a fairly precise significance, and this more particularly when, as here, renal disease and diabetes can be excluded from the diagnosis. In such circumstances the suggestion of some serious blood disease is most direct and strong, and of these pernicious anæmia is perhaps the most frequent, and certainly is the one most apt to be overlooked. It may, indeed, be fairly said that in practical experience, and given the absence of renal disease and of diabetes, the recognition of multiple retinal hæmorrhages in each fundus oculi is most frequently associated with pernicious anæmia. The enlargement of the spleen or lymphatic glands, or of both of these, in other blood diseases, such as leucocythæmia and Hodgkin's disease, makes an error in diagnosis in such conditions less probable. But in pernicious anæmia the gradual movement of the disease and the absence of physical signs, using this term in its conventional sense, are apt to mislead the observer and to cause the diagnosis to be missed. Hence the great advantage of recognising that in this disease multiple retinal hæmorrhages are of frequent occurrence. It cannot be claimed that they are present in every case, but they are a prominent fact in the great majority of cases, and were the ophthalmoscope used as a routine procedure the disease would most certainly be less frequently overlooked. No doubt, as already stated, retinal hæmorrhages occur in renal disease, in diabetes, and in such blood affections as leucocythæmia, etc. But these conditions are not likely to be confused with pernicious anæmia by any careful observer, and, given proof of their absence, the existence of retinal hæmorrhages raises the probability of pernicious anæmia to a high level.

Further, the retinal hæmorrhages of pernicious anæmia afford assistance in distinguishing this disease from certain other conditions with which it is more or less apt to be confused. One of these is malignant disease, another, severe chlorosis, and a third, anæmia secondary to loss of blood. Not a few cases of malignant disease, and more particularly of malignant disease of the stomach, have undoubtedly been regarded as examples of pernicious anæmia until the discovery of a tumour, either later in the history of the case or on post-mortem examination, has demonstrated the correct diagnosis. Indeed, though the statement is rather of the rough-and-ready order, it is not without value to repeat that when a patient is manifestly the subject of some serious illness having a more or less chronic course, extending, say, over several weeks or months, and not explained by evidences of visceral disease, the probability of either pernicious anæmia or malignant disease should be carefully remembered. The point of special interest here is that, in the circumstances just related, the existence of multiple retinal hæmorrhages, as in our present patient, strongly inclines the diagnosis in the direction of pernicious anæmia. With regard to chlorosis and to the anæmia following hæmorrhage, it may be accepted that in these conditions retinal hæmorrhages are exceptional, and that even when present they are generally both few in number and insignificant in size. Thus the position is reached that retinal hæmorrhages occurring in association with such general symptoms as anæmia, progressive weakness, and breathlessness, and apart from renal disease, from diabetes, and from enlargements of the spleen and lymphatic glands, are usually, if not invariably, an evidence of pernicious anæmia. Malaria may perhaps be mentioned as a general disease in which mutliple retinal hæmorrhages are occasionally seen, but cases of malaria are infrequent in this country, and the clinical history and examination of the blood are sufficient, other facts apart, to establish the diagnosis.

Here, then, is a patient who is presented as the subject of an œdema existing apart from visceral disease. Such a conjunction of events has compelled us to consider the possibility of (1) peripheral neuritis, and (2) of some serious depreciation of the quality of the blood. Of the first of these there is no evidence—no anæsthesia, no muscular weakness or atrophy, no tenderness of the muscles or nerve trunks, no ataxia, and no deficiency of the knee-jerks. On the other hand, the patient is certainly anæmic, and ophthalmoscopic examination displays a picture which, in the circumstances, is in the highest degree suggestive of a recognised blood disease, namely, pernicious anæmia. Naturally, therefore, the next step in the investigation of the case was an examination of the blood, and when this was undertaken, all possibility of doubt disappeared. The red corpuscles were found to be reduced to 31 per cent. of the normal, and the hæmoglobin to 41 per cent., the greater reduction of the corpuscles than of the hæmoglobin indicating a high colour index (1.3), and being, as is well known, a characteristic feature of the pernicious type of anæmia. In addition, the red cells varied much in size, many were conspicuously deformed or altered in shape (poikilocytosis), and a few nucleated red cells were seen. The white corpuscles numbered only 1,200 per cubic mm.—that is, they were far below the normal count, and this condition of leucopænia, in association with a marked reduction of the red cells, with the presence of nucleated reds, and with a high colour index, is highly suggestive of pernicious anæmia.

It is not unimportant to state the position in the fashion just adopted, for the blood examination should never be exalted to an absolutely conclusive position. With such a combination of blood conditions as exists in the present case the suggestion of pernicious anæmia certainly reaches a very high level. But the level is hardly one of absolute certainty. And, on the other hand, it must be remembered that in some stages and phases of pernicious anæmia the blood may give no positive evidence to support the diagnosis. All the various aspects of the case, and not merely the blood examination, must be brought under review, and in particular it is necessary to make more than one examination of the blood, and this especially when the first examination gives only negative results. Probably too much stress is often placed on the condition of the blood in the diagnosis of pernicious anæmia, and it is well to emphasise the necessity of keeping the facts so obtained in their proper position and due perspective.

Reviewing, then, the present case, it may be said that its special interest resides in the fact that its leading clinical feature, namely œdema of the lower limbs, is discovered on examination to be an expression of pernicious anæmia, and that this symptom was so conspicuous at a date comparatively early in the history of the case that the existence of anæmia as its cause was overlooked. This is by no means an exceptional experience, and hence it is well that we should take note of the practical lesson which it illustrates.

There is another fact in the clinical record which is also of some considerable diagnostic importance—namely, a certain degree of pyrexia. When first seen in the out-patient department the patient was found to have a temperature of 100.6° F., and though within a few days after patient's admission to the hospital the temperature had fallen to the normal level, there were on several occasions during her residence records approaching or reaching 100° F. Now this is a common experience both in pernicious anæmia and in certain other affections of the blood, and it is of distinct value in establishing the diagnosis. Occasionally, too, the temperature in pernicious anæmia is a commanding feature of the case. It may reach 103° or 104°, or even higher, and exceptionally these high records continue more or less persistently for several weeks. In this is to be found the explanation of the ease with which now and again a case of pernicious anæmia receives a diagnosis of enteric fever; and the mistake is the more readily made when, as is by no means infrequent, diarrhœa and enlargement of the spleen are numbered among the clinical evidences of the disease. A febrile temperature without physical evidences of any disease competent to explain it is one of the common problems of medical practice. Such an experience necessarily gives rise to suspicions of enteric fever, of tuberculosis, or of some form of septicæmia. It is well to add pernicious anæmia and other more or less closely allied disorders of the blood to this list of the possible causes of pyrexia existing apart from physical signs of disease. And when this is remembered, a certain number of cases of unexplained pyrexia will be readily and accurately interpreted. Unlike the œdema, the existence of pyrexia can hardly be regarded as directly due to the anæmia. Its existence in our present patient, and to a still more marked degree in many other patients, goes therefore to support the view that pernicious anæmia is a disease due to some toxic agent exerting a prejudicial influence, not merely on the blood, but on many other tissues. Hence many of the phenomena of the disease—for example, occasional or persisting pyrexia, vomiting, diarrhœa, enlargement of the spleen, which are difficult to explain as consequences of anæmia, become readily understood; they appear, equally with the anæmia, as direct and immediate effects of the primary toxic agent—an agent which Dr. Wm. Hunter regards as one possessing a definite and specific character.

The suggestion or supposition that pernicious anæmia is essentially a toxæmia has, of course, important applications in reference to the treatment of the disease, but for the moment I want rather to emphasise its relation to diagnosis. Manifestly, on such a view, it is conceivable that while in one case the blood may suffer the main damage—in other cases, the stress of the disease may fall on other tissues, with the result that vomiting, diarrhœa, temperature disturbance, splenic enlargement, etc., may be more or less conspicuous clinical features. In such circumstances the physician's attention is, as it were, directed away from the anæmia—which, by the way, is not invariably and in all stages of the disease of an extreme type—and thus the true nature of the case may be missed. Hence the fact of pyrexia in our present patient not only helps the diagnosis in this individual instance, but serves also to press on our attention the more modern view of the pathology of pernicious anæmia. And this view, leaving for the moment the question of its accuracy on one side, has the practical advantage that it prepares us to find in cases of pernicious anæmia signs and symptoms other than those which are merely secondary to the state of anæmia; it also lessens the risk of an error in diagnosis, when, as sometimes happens, such signs and symptoms form the prominent clinical features of the case. In our present patient, the œdema, and to some extent the pyrexia, seem to have attracted attention, and to have obscured, though perhaps not quite justifiably, the importance of the anæmia; in other instances marked and persisting temperatures or severe gastro-intestinal symptoms have produced a similar result.

There is yet one further group of symptoms which may be present in pernicious anæmia and which, by their relative prominence, may cause the true diagnosis to be overlooked. I mean symptoms of nervous disturbance. It is well known that pernicious anæmia is in occasional instances associated with a combined sclerosis of the posterior and lateral columns of the spinal cord, and there is evidence also that it may be associated with peripheral neuritis. Hence a case of pernicious anæmia may appear with definite signs of organic nervous disease; and here again, unless the practitioner is alive to the possibilities of the situation, the case may be seriously misread. But, rather than these comparatively conspicuous changes, I want to emphasise the commanding position which pain, and especially abdominal pain, may occupy in the symptomatology of pernicious anæmia. This point is not illustrated in our present patient, but it may be regarded as appropriate to our discussion as it is one of the facts responsible at times for failure to recognise a case of pernicious anæmia. The pain is usually at the epigastrium, but it may extend over the whole abdomen, and more particularly when, as is not uncommon, vomiting is a feature in the case, the condition is apt to be mistaken for some form of dyspepsia; in occasional instances hæmatemesis occurs, and thus provides a further opportunity for misreading the diagnosis. Still another feature of pernicious anæmia which may lead to a suspicion of serious disease of the stomach is the absence of hydrochloric acid from the gastric juice. For the most part the abdominal pain or other form of discomfort is far from being severe, but in other cases it is extreme, and may even be described as agonising; in one instance I have known the abdomen opened on the view, suggested by paroxysmal attacks of severe pain, that the case was probably one of biliary or pancreatic calculus; Dr. Hunter speaks of "acute gastric discomfort" and of "attacks of tearing, griping pain in the intestine," and Dr. J. George Taylor has described an instance in which the pain "came on rather suddenly, lasted for several hours, and appeared to cause intense agony." Therefore abdominal pain, even of a severe order, must be included among the possible events which find an explanation in the diagnosis of pernicious anæmia.

To sum up the clinical experience of this individual case, the problem was to find an explanation of a considerable and long-standing œdema of the lower limbs. Examination of the chest, abdomen, pelvis, and urine proving negative, the possibility of peripheral neuritis or of some disease of the blood came into view. As the usual tests of the nervous apparatus gave normal results, peripheral neuritis was excluded, and the condition of the blood was brought acutely under suspicion. This suspicion was deepened by the discovery of multiple retinal hæmorrhages, and was confirmed by an examination of the blood itself. Thus the case is correctly presented as one of pernicious anæmia associated with considerable subcutaneous œdema, retinal hæmorrhages, and a moderate degree of pyrexia. And perhaps its principal value as a clinical study is the illustration it affords of one of the fashions in which, in consequence of the prominence assumed by an individual symptom, the anæmia may be pushed into the background, and thus escape the attention of the clinical observer.

